# Predicted 25-hydroxyvitamin D in relation to incidence of breast cancer in a large cohort of African American women

**DOI:** 10.1186/s13058-016-0745-x

**Published:** 2016-08-12

**Authors:** Julie R. Palmer, Hanna Gerlovin, Traci N. Bethea, Kimberly A. Bertrand, Michael F. Holick, Edward N. Ruiz-Narvaez, Lauren A. Wise, Stephen A. Haddad, Lucile L. Adams-Campbell, Harvey W. Kaufman, Lynn Rosenberg, Yvette C. Cozier

**Affiliations:** 1 Slone Epidemiology Center at Boston University, 1010 Commonwealth Avenue, 4th floor, Boston, MA 02215 USA; 2 Department of Biostatistics, Boston University School of Public Health, Boston, MA 02118 USA; 3 Department of Medicine, Physiology and Biophysics, Boston University School of Medicine, Boston, MA 02118 USA; 4 Department of Epidemiology, Boston University School of Public Health, Boston, MA 02118 USA; 5 Georgetown University Lombardi Cancer Center, Washington, DC, 20057 USA; 6 Quest Diagnostics, Inc, Madison, NJ 07940 USA

**Keywords:** Breast cancer, Vitamin D, African American, Prediction models

## Abstract

**Background:**

Vitamin D deficiency, which has been linked to an increased risk of colorectal cancer, is particularly common among African Americans. Previous studies of vitamin D status and breast cancer risk, mostly conducted in white women, have had conflicting results. We examined the relationship between predicted vitamin D status and incidence of breast cancer in a cohort of 59,000 African American women.

**Methods:**

Participants in the Black Women’s Health Study have been followed by biennial mail questionnaires since 1995, with self-reported diagnoses of cancer confirmed by hospital and cancer registry records. Repeated five-fold cross-validation with linear regression was used to derive the best 25-hydroxyvitamin D (25(OH)D) prediction model based on measured 25(OH)D in plasma specimens obtained from 2856 participants in 2013–2015 and questionnaire-based variables from the same time frame. In the full cohort, including 1454 cases of incident invasive breast cancer, Cox proportional hazards models were used to compute the incidence rate ratio (IRR) for each quartile of predicted vitamin D score relative to the highest quartile. Predicted vitamin D score for each two-year exposure period was a cumulative average of predicted scores from all exposure periods up to that time.

**Results:**

Twenty-two percent of women with measured 25(OH)D were categorized as “deficient” (<20 ng/mL) and another 25 % as “insufficient” (20–29 ng/mL). The prediction model explained 25 % of variation in measured 25(OH)D and the correlation coefficient for predicted versus observed 25(OH)D averaged across all cross-validation runs was 0.49 (SD 0.026). Breast cancer risk increased with decreasing quartile of predicted 25(OH)D, *p* for trend 0.015; the IRR for the lowest versus highest quartile was 1.23 (95 % confidence interval 1.04, 1.46).

**Conclusions:**

In prospective data, African American women in the lowest quartile of cumulative predicted 25(OH)D were estimated to have a 23 % increased risk of breast cancer relative to those with relatively high levels. Preventing vitamin D deficiency may be an effective means of reducing breast cancer incidence in African American women.

## Background

Vitamin D is a precursor to 1,25-dihydroxyvitamin D (1,25(OH)2D), a steroid hormone that mediates numerous actions in the body, including pathways involved in cancer. Mechanisms underlying the possible anticancer effects of 1,25(OH)2D include induction of apoptosis, stimulation of differentiation, anti-inflammatory effects, anti-proliferative effects, and inhibition of angiogenesis, invasion, and metastasis [1]. Substantial evidence from epidemiologic studies links higher vitamin D status to a reduced risk of colon cancer [2, 3]. However, results from studies of vitamin D status and breast cancer risk have been conflicting, with several prospective studies finding no association with circulating levels of 25-hydroxyvitamin D (25(OH)D) [4–7]. There has been little research on the relationship between vitamin D status and risk of breast cancer in African Americans [8–11]. Vitamin D deficiency (<20 ng/mL) or insufficiency (20 to 29 ng/mL) is common in African Americans, in large part due to darker skin pigmentation on average, which reduces the penetration of sunlight and subsequent production of vitamin D_3_ in the skin [12, 13].

Each of the study designs used for assessment of the relationship between vitamin D status and risk of breast cancer has drawbacks. Case-control studies of plasma or serum 25(OH)D levels are prone to reverse causality because blood specimens are usually drawn around the time of breast cancer surgery, at which point 25(OH)D levels may have been affected by the disease process or patients may have changed their habits regarding time spent outdoors, diet, and supplementation.

Prospective cohort studies with pre-diagnostic blood specimens overcome the problem of reverse causality. However, they typically have only a single blood draw for each participant, representing exposure status at only one point in time, whereas evidence suggests that 25(OH)D levels vary over time depending upon season, age, weight, and other characteristics [14, 15]. Studies of dietary intake or use of vitamin D supplements may have measures at more than one time point, but these measures do not take into account sun exposure and skin pigmentation, which also influence blood levels of 25(OH)D [16]. These limitations may be overcome by use of predicted vitamin D status, with updating and averaging of the predicted values over time. This method was first demonstrated by Giovannucci et al. in one of the initial studies to show an association between vitamin D status and colon cancer [2].

While predicted 25(OH)D will be imprecise for any given individual, it can be effective for ranking study participants into disparate categories of exposure, such as lowest quartile vs. highest quartile of predicted value. The validity of this method will depend on the specimens used for establishing the prediction model, collection of data on the important determinants of 25(OH)D levels in the study population, and availability of repeated measures of those determinants over time.

We used prospectively collected data and blood specimens from 2856 study participants in the Black Women’s Health Study to develop a prediction model for 25(OH)D. We then assessed the relationship between predicted vitamin D status and risk of breast cancer in the entire cohort.

## Methods

### Study population

The Black Women’s Health Study (BWHS) began in 1995 when 59,000 African American women aged 21–69 years from across the USA completed mailed health questionnaires. Participants have completed follow-up questionnaires every two years. Follow-up was complete through 2013 for over 85 % of person-time since 1995. The Institutional Review Board of Boston University approved the protocol and reviewed the study annually.

At baseline, participants were asked about use of vitamin D supplements, use of multivitamins, weight, height, number of births, timing of each full-term birth, lactation, age at menarche, use of oral contraceptives, breast cancer in first-degree relatives, vigorous physical activity, alcohol consumption, cigarette smoking, menopausal status, age at menopause, use of supplemental female hormones, years of education, and many other factors. The biennial follow-up questionnaires ascertained occurrences of incident breast cancer and updated information on use of vitamin D supplements and multivitamins, and most other variables. A modified version of the NCI-Block food frequency questionnaire was used to ascertain usual diet in 1995 and 2001 [17].

### Breast cancer cases

Each BWHS questionnaire asks about new diagnoses of breast cancer and the year of diagnosis. Participants are contacted for permission to obtain pathology reports and other medical records and data are also obtained from state cancer registries in the 24 states in which 95 % of participants live. We were able to obtain medical records, cancer registry records, or both for approximately 95 % of women who reported incident breast cancer, of which 99 % were confirmed. Only cases of confirmed incident breast cancer were included in the present analysis. In the early years of the study, 1995–2000, testing for estrogen receptor (ER) and progesterone receptor (PR) was not universal, and thus we have missing data on ER and PR status for some participants. Among cases with known status, the proportions with ER+/PR+, ER+/PR-, ER-/PR+, and ER-/PR- tumors are 50 %, 14 %, 2 %, and 34 %, respectively, similar to the distributions observed for African American women in the SEER registry and other population-based data [18–20]. In previous comparisons of cases with data on receptor status to cases with unknown receptor status, the two groups were similar with regard to the prevalence of known breast cancer risk factors [21].

### Blood collection and laboratory assays

Collection of blood specimens from BWHS participants began in 2012 and will continue through 2017, by which time all living study participants will have had an opportunity to provide a sample. Of participants approached to date, about 25 % have provided samples. Participants are mailed an informed consent, explanatory materials, a pre-printed laboratory requisition form, and instructions for locating a nearby blood collection site. Blood specimens are collected and tested by Quest Diagnostics (Madison, NJ, USA) an accredited national clinical laboratory [22, 23]. Liquid chromatography-tandem mass spectrometry (LC-MS/MS) was used for measurement of 25(OH)D [24, 25], which was carried out at three Quest central laboratories. National Institute of Standards and Technology Standard Reference Material for 25(OH)D in human serum (NIST SRM 972) was used for quality control. Written informed consent to use the blood samples for health-related research for the entirety of the study was obtained from participants who provided samples.

### 25(OH)D prediction model

Development of the prediction model was based on 25(OH)D values obtained from assays of plasma samples provided between 2013 and early 2015, the period immediately following completion of the 2013 BWHS questionnaire. During that period, 3539 participants provided blood samples with signed informed consent. We excluded 276 women with prevalent cancer at the time of the blood draw and 407 who had missing data on any of the candidate predictors of 25(OH)D, for an analytic sample of 2856. Variables that were known or suspected to be related to endogenous levels of 25(OH)D and for which data were available from the 2013 questionnaire were considered as possible predictors: use of vitamin D supplements (with or without calcium, at least twice a week), multivitamin use (at least twice a week, not specified whether they included vitamin D or how much), body mass index (BMI, kg/m^2^) (considered as both a categorical and a continuous variable), vigorous exercise, walking for exercise, current cigarette smoking, current alcohol consumption, use of female hormones, use of oral contraceptives, and menopausal status. Dietary intake of vitamin D derived from responses to a modified version of the NCI-Block food frequency questionnaire completed in 2001 was also considered as a predictor. A solar UV-B flux variable (high, medium, low levels of solar UV-B radiation) was created as a proxy for ambient sun exposure based on state of residence in 2013 and the reported average annual UV-B radiation in each state [26], and considered for the prediction model.

Repeated k-fold cross-validation was used to derive the best 25(OH)D prediction model [27]. The 2856 specimens were divided into five groups of equal size, with 4/5 serving as a training set and the remaining 1/5 serving as the test set. Using the ‘caret’ package in R [28], stepwise selection by Akaike’s information criterion was performed on each training set to identify the optimal predictors in a generalized linear model for continuous 25(OH)D. The best fit model parameters were then used to predict 25(OH)D in the test set, at which point Pearson’s correlation coefficient was computed for the linear association between observed and predicted 25(OH)D values. This procedure was performed another four times with each remaining 1/5 serving as the test set. We repeated this 100 times, with the full sample divided into a different set of five groups each time. Overall model prediction performance was calculated as the average R-squared value across the 100 repetitions and each of the five folds. The generalized linear models were adjusted for season of blood draw, laboratory, and age, for the purpose of controlling variability when estimating beta coefficients for predictors.

Baseline data from the 1995 BWHS questionnaire were then used in combination with beta-coefficients for each of the predictors to compute a predicted 25(OH)D level at baseline in 1995 for the entire BWHS cohort. Participants were excluded from the analyses if they had missing data on any of the predictors at baseline. Predicted 25(OH)D level was then updated every two years using the same beta coefficients and new values of the predictors. If data were missing for a given variable at some point during follow-up, the value from the previous cycle was carried forward. We then computed a cumulative average of predicted 25(OH)D. The cumulative average method [29, 30] has been used previously in vitamin D prediction models and for exposures such as dietary intake and physical activity [2, 31, 32]. For this approach, the predicted 25(OH)D score for a given time point is the average of scores from previous time points up to and including that time point. This method may better represent average long-term vitamin D status over the period of follow-up for each individual [33].

### Statistical analysis of 25(OH)D in relation to breast cancer risk

Analyses of the association between predicted vitamin D status and breast cancer risk included all BWHS participants who had not been diagnosed with cancer prior to enrollment in the cohort and had complete information from the baseline questionnaire on each of the variables included in the 25(OH)D prediction model. Each participant contributed person-time from baseline in 1995 until diagnosis of breast cancer, death, loss to follow-up, or end of follow-up in 2013, whichever came first. Predicted 25(OH)D status was analyzed in quartiles, with highest quartile as the reference category. We used Cox proportional hazards regression, stratified by age (year) and questionnaire cycle (two years) to estimate the incidence rate ratio (IRR) and 95 % confidence interval (CI) for quartile of predicted 25(OH)D in relation to breast cancer incidence, with adjustment for number of births (0, 1, 2, ≥3), age at first birth (<20, 20–24, ≥25), age at menarche (<11, 12–13, ≥14 years), age at menopause (<45, 45–49, ≥50 years, or premenopausal), first-degree family history of breast cancer (yes, no), recent oral contraceptive use (within the previous 5 years), long-term oral contraceptive use (≥10 years), duration of use of estrogen with progesterone postmenopausal hormones (≥5 years), and BMI (<25, 25–29, 30–34, ≥35 kg/m^2^). Covariates that changed over time were treated as time-dependent. In addition to the overall analyses, we conducted analyses separately for ER+ and ER- breast cancer and within strata of age (<45 years and ≥45 years) and current use of vitamin D supplements (yes, no).

## Results

### Predicted 25(OH)D

**Fig. 1:**
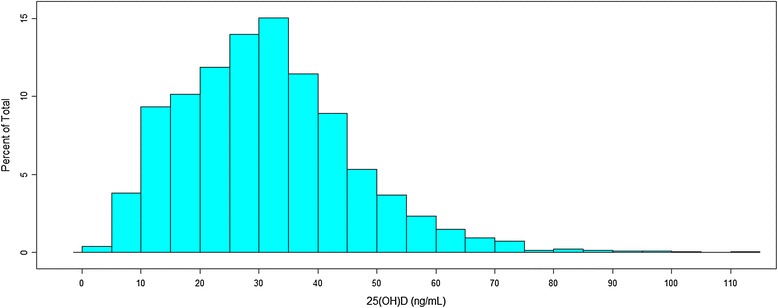
Measured plasma 25-hydroxyvitamin D (*25(OH)D*) (ng/mL) among 2856 participants in the Black Women’s Health Study

Figure 1 displays the frequency distribution of measured plasma 25(OH)D in the 2856 specimens that were included in model development. Quartiles of measured plasma 25(OH)D had the following cut points: 21 ng/mL, 31 ng/mL, and 40 ng/mL. Overall, 22 % of specimens had plasma 25(OH)D levels <20 ng/mL (a commonly used cut-point for deficiency) and 47 % had a value <30 ng/mL (cut-point for insufficiency) [34]. Among women who did not report taking a vitamin D supplement, 34 % had <20 ng/mL and 64 % had <30 ng/mL of plasma 25(OH)D.

Table 1 shows the variables retained in the prediction model. Beta-coefficients for age, season of blood draw, and UV-B flux, which were included as adjustment factors but not used in the derivation of predicted vitamin D status, are also given in Table 1. The strongest predictor, as indicated by squared semi-partial correlation coefficients, was vitamin D supplementation, which independently accounted for 10 % of the total variation in the observed vitamin D levels after adjustment for the other retained predictors in the model. Multivitamin use, dietary intake, physical activity, use of female hormones, and use of oral contraceptives were associated with higher levels of predicted 25(OH)D, whereas cigarette smoking, alcohol consumption, and higher BMI were associated with lower levels. Overall, the model was estimated to explain 25.2 % of variation in 25(OH)D. The correlation coefficient for predicted vs. observed 25(OH)D averaged across all cross-validation runs was 0.49 (SD 0.026). On average, across the 100 repetitions of five-fold cross-validation, 40 % of the testing set participants were classified into the same quartile for observed and predicted (agreement diagonal) and 82 % were classified in either the same or an adjacent quartile.

**Table 1:** Predictors of plasma 25-hydroxyvitamin D (25(OH)D) in 2856 participants from the Black Women’s Health Study

Predictors	Beta-coefficient for difference in 25(OH)D (ng/mL)	*P*	Squared semi-partial correlation coefficient (%)^a^
Intercept	11.94		
Supplementary vitamin D	9.66	<0.0001	10.41
Multivitamin use	4.60	<0.0001	2.75
Dietary vitamin D (kcal-mcg/day)	0.33	0.01	0.23
Body mass index			
25.0–29.9	-2.21	0.001	0.22
30.0–34.9	-2.96	<0.0001	0.36
≥ 35.0	-4.71	<0.0001	0.84
Postmenopausal hormone use			
Past use ≥5 years	2.70	0.001	0.30
Current use <5 years	2.39	0.02	0.18
Current use ≥5 years	2.31	0.03	0.16
Vigorous physical activity, ≥1 hour/week	1.59	0.001	0.34
Alcohol consumption			
1–6 drinks per week	-2.28	0.02	0.20
≥7 drinks per week	-2.97	0.006	0.26
Cigarette smoking	-1.58	0.13	0.08
Recent use of oral contraceptives	2.58	0.003	0.28
Use of oral contraceptives ≥10 years	1.60	0.02	0.19
Variables controlled in the regression models			
Season of blood draw			
Summer	1.66	0.01	0.14
Winter	-1.88	0.003	0.28
UVB flux 160+	-1.93	0.01	0.09
Age (years)	0.24	<0.0001	1.40

^a^Attributable proportion of variation in 25(OH)D explained by parameter, after adjustment for the other predictors in the model

Adjusted R-squared = 0.252

### Association between predicted 25(OH)D and incidence of breast cancer

A total of 1454 cases of incident invasive breast cancer were identified during follow-up from 1995 through 2013, including 433 ER- cases, 802 ER+ cases, and 219 cases with unknown ER status.

Women in the lowest quartile of predicted 25(OH)D had an increased risk of breast cancer: the IRR for the lowest vs. highest quartile (reference) was 1.06 (95 % CI 0.92, 1.23) in analyses adjusted for age and period and 1.23 (95 % CI 1.04, 1.46) in multivariable analyses (Table 2). The strongest confounder was pre-diagnostic BMI; higher BMI was associated with lower levels of measured 25(OH)D and, in this dataset, with lower incidence of breast cancer.

**Table 2:** Cumulative predicted vitamin D status in relation to breast cancer incidence, overall and by estrogen receptor (ER) status of the breast tumor

Predicted 25(OH)D (quartiles)	Breast cancer cases	Person-years	Age + period IRR	95 % CI	MV IRR^a^	95 % CI	*P* trend
All cases							
4 (highest)	338	183,614	1.00	Ref	1.00	Ref	0.015
3	351	183,625	1.02	0.88, 1.19	1.08	0.93, 1.26	
2	365	183,603	1.02	0.88, 1.18	1.12	0.95, 1.31	
1 (lowest)	400	183,568	1.06	0.92, 1.23	1.23	1.04, 1.46	
ER- cases							
4 (highest)	109	183,326	1.00	Ref	1.00	Ref	0.42
3	100	183,346	0.90	0.68, 1.18	0.94	0.71, 1.25	
2	109	183,336	0.93	0.71, 1.21	1.02	0.77, 1.36	
1 (lowest)	115	183,324	0.94	0.72, 1.22	1.12	0.82, 1.52	
ER+ cases							
4 (highest)	182	183,430	1.00	Ref	1.00	Ref	0.035
3	192	183,446	1.05	0.86, 1.29	1.12	0.91, 1.38	
2	215	183,403	1.13	0.93, 1.38	1.25	1.01, 1.55	
1 (lowest)	213	183,415	1.08	0.88, 1.32	1.26	1.00, 1.58	

^a^Adjusted for age (continuous), family history of breast cancer, age at menarche, age at menopause, parity, age at first birth, oral contraceptive use, body mass index, and use of estrogen and progesterone female hormones

*25(OH)D* 25-hydroxyvitamin D, *IRR* incident rate ratio, *MV* multivariate model, *CI* Confidence interval

There was evidence of a linear trend toward increasing risk with decreasing quartile of predicted 25(OH)D (*P* trend = 0.015). The association between predicted 25(OH)D and ER+ breast cancer was similar to that for breast cancer overall, with a multivariable-adjusted IRR for lowest vs. highest quartile of 1.26 (95 % CI 1.00, 1.58). For ER- breast cancer, the IRR was 1.12 (95 % CI 0.82, 1.52) for the same comparison.

In age-specific analyses (Table 3), IRRs for the lowest versus highest quartiles of predicted 25(OH)D were 1.28 (95 % CI 0.90, 1.82) in women younger than 45 years and 1.25 (95 % CI 1.03, 1.51) in older women. The observed association was present regardless of vitamin D supplementation: IRRs for the lowest vs. highest quartiles of predicted score were 1.23 (95 % CI 1.02, 1.49) among non-users and 1.25 (95 % CI 0.82, 1.90) among users. Results were inconsistent across strata of BMI. Among women who were obese at baseline, a strong significant association was observed, with an IRR of 1.65 (95 % CI 1.25, 2.19) for lowest relative to highest quartile, whereas among overweight and normal weight women, IRRs for the same comparisons were 1.04 (95 % CI 0.80, 1.35) and 1.09 (95 % CI 0.87, 1.45), respectively. However, there was not a statistically significant interaction (*P* interaction = 0.18).

**Table 3:** Cumulative predicted vitamin D status in relation to breast cancer incidence, within strata of age and vitamin D supplement use

Predicted 25(OH)D (quartiles)	Breast cancer cases	Person-years	MV^a^ IRR	95 % CI	*P* trend
Age <45 years					
4 (highest)	86	89,683	1.00	Ref	0.20
3	100	89,686	1.20	0.89, 1.62	
2	98	89,675	1.19	0.87, 1.63	
1 (lowest)	107	89,651	1.28	0.90, 1.82	
Age ≥45 years					
4 (highest)	262	93,921	1.00	Ref	0.045
3	275	93,922	1.13	0.95, 1.35	
2	254	93,949	1.09	0.91, 1.31	
1 (lowest)	272	93,924	1.25	1.03, 1.51	
BMI^b^ <25 kg/m^2^					
4 (highest)	126	72,829	1.00	Ref	0.99
3	112	72,853	0.98	0.75, 1.27	
2	130	72,830	1.13	0.87, 1.46	
1 (lowest)	133	72,824	1.09	0.83, 1.42	
BMI^b^ 25 to <30 kg/m^2^					
4 (highest)	133	57,686	1.00	Ref	0.89
3	126	57,701	1.03	0.80, 1.32	
2	114	57,708	0.96	0.75, 1.25	
1 (lowest)	121	57,708	1.04	0.80, 1.35	
BMI^b^ ≥30 kg/m^2^					
4 (highest)	108	53,074	1.00	Ref	0.0003
3	109	53,078	1.19	0.91, 1.56	
2	115	53,065	1.36	1.04, 1.79	
1 (lowest)	127	53,054	1.66	1.25, 2.19	
No vitamin D supplementation					
4 (highest)	270	156,422	1.00	Ref	0.048
3	302	156,416	1.10	0.93, 1.30	
2	297	156,415	1.06	0.89, 1.27	
1 (lowest)	338	156,372	1.23	1.02, 1.49	
Vitamin D supplementation, yes					
4 (highest)	53	27,203	1.00	Ref	0.31
3	60	27,206	1.13	0.77, 1.65	
2	64	27,201	1.14	0.77, 1.70	
1 (lowest)	70	27,176	1.25	0.82, 1.90	

^a^Adjusted for age (continuous), family history of breast cancer, age at menarche, age at menopause, parity, age at first birth, oral contraceptive use, body mass index (BMI), and use of estrogen and progesterone female hormones. ^b^Analyses stratified by baseline BMI group were also adjusted for continuous BMI

*25(OH)D* 25-hydroxyvitamin D, *IRR* incident rate ratio, *MV* Multivariate model, *CI* Confidence interval

When the analyses were repeated using a simple update of predicted 25(OH)D instead of a cumulative average, the results were essentially the same: the IRR for lowest quartile to highest quartile for all breast cancer was 1.23 (95 % CI 1.04, 1.44).

## Discussion

To our knowledge, this is the first study to use a 25(OH)D prediction model to assess the relationship between vitamin D status and incidence of breast cancer. Women in the lowest quartile of predicted 25(OH)D over the course of follow-up were estimated to have a 23 % increased risk of breast cancer compared with those in the highest quartile. Based on the distribution of measured plasma 25(OH)D levels in the 2856 specimens that formed the basis of the prediction model, almost all women in the lowest quartile would have had a level considered deficient, and all women in the highest quartile would have had levels considered sufficient. In analyses stratified by BMI, a significant positive association was observed only among obese women.

Most previous studies of vitamin D status in relation to breast cancer risk used serum or plasma levels of 25(OH)D from a single point in time as the measure of vitamin D exposure. Higher levels were associated with lower risk in a number of case-control studies [35, 36]. The majority of prospective cohort studies have yielded null results [35, 36] but a significant inverse association was observed between 25(OH)D levels and overall breast cancer risk in the E3N, a large prospective cohort study from France [37]. Three of the largest prospective studies – the European Prospective Investigation into Cancer and Nutrition [7], the Nurses’ Health Study II [5], and a combined analysis of the NYU Women’s Health Study and the Swedish Mammography Cohort [6] - found no association between 25(OH)D levels and overall breast cancer risk. However, inverse associations were observed in some subgroups. The NYU/Swedish analysis observed an inverse association among women under age 45 years and among premenopausal women [6]. In the Multiethnic Cohort Study, higher levels of plasma vitamin D were associated with a significant reduction in breast cancer incidence in white women but not in other racial/ethnic groups [10]. In the Nurses’ Health Study, there with a statistically significant inverse trend across quintiles among women aged 60 years and older, but no trend among younger women [38]. In the present study, IRRs were similar across different ages.

In the Nurses’ Health Study II, there was a significant interaction with BMI, with a strong positive interaction observed among women in the highest category of BMI (≥25) but no association among women with BMI <25 [5]. Our BMI-stratified analyses produced similar findings, with a positive association observed among women with BMI ≥30, but not among women with lower BMIs; we were able to examine three strata of BMI because of the greater number of breast cancer cases and the higher prevalence of obesity. Other studies have reported no interaction with BMI [37–40]. The reasons for this interaction, if not due to chance, are unclear.

Although findings from basic research suggest that vitamin D may have a greater impact on ER+ breast cancer than ER- breast cancer through attenuation of estrogen signaling and synthesis, [41–43], previous investigations that assessed ER+ and ER- breast cancer have not found a stronger association for ER+ breast cancer. In the Nurses’ Health Study [38] and two other studies [44, 45], there was evidence of a stronger association with ER- breast cancer, but most findings have been consistent across subtypes. In the present study, there was significant association with incidence of ER+ breast cancer, and a weaker association with ER- cancer.

Circulating levels of 25(OH)D change over time, with a single measurement reflecting vitamin D from dietary and solar sources within a three-week half-life [46]. Since most observational studies have relied on plasma 25(OH)D measured from a single blood draw, often taken many years before the diagnosis of breast cancer, non-differential misclassification with bias towards the null is likely. The reproducibility of 25(OH)D measurements obtained at two time points has been examined in a few studies. In most instances, including among African American participants in the Southern Community Cohort Study [47], specimens taken 1–2 years apart were strongly correlated, but correlation diminished after longer intervals since first blood draw. Thus, 25(OH)D levels from a single blood draw appear to be valid measures of usual levels within two years, but no inference can be made about levels more removed in time. An important strength of using a vitamin D prediction model is the use of repeated measures to estimate vitamin D levels at multiple time points. In the present study, we were able to calculate predicted 25(OH)D at baseline in 1995 and every two years afterwards through the end of follow-up. We created a cumulative average exposure variable, which represented an average predicted level for each participant from baseline through the end of her follow-up. A cumulative average of predicted 25(OH)D is likely to be a better proxy for extremes of vitamin D status in the years prior to breast cancer diagnosis compared with a single blood draw.

Other studies have assessed intake of foods containing vitamin D or intake of vitamin D supplements in relation to breast cancer risk. Several studies found reduced risk of breast cancer among women who took supplements or were in the highest categories of dietary intake [48–50], whereas others found no association [51–57].

A notable strength of the present study is the k-fold cross-validation method used to develop and test the prediction model. Most previous vitamin D status prediction models have been based on model development in a single training set with testing in a single test set [2, 33, 58]. Our machine-learning approach repeated the training and testing steps 100 times, each time using a different subset of the sample for each step. The validity of our model was evidenced by the high correlation coefficient, 0.49. Bertrand et al. developed and tested 25(OH)D prediction models in similarly sized samples from Nurses’ Health Study, Nurses’ Health Study II, and Health Professionals Follow-Up Study and reported correlation coefficients of 0.33, 0.42, and 0.30, respectively [33].

Prediction models for vitamin D status may perform better in populations with African ancestry than in populations with European ancestry because sun exposure, which is difficult to quantify, contributes less to 25(OH)D levels among African Americans, because on average persons of African ancestry have darker skin pigmentation. Thus, variables such as use of vitamin D supplements, BMI, and cigarette smoking, will tend to be more important predictors in an African American population. In a study of circulating 25(OH)D levels in African American and white participants from a nationwide study of radiologic technologists, UV radiation factors (e.g., time spent outdoors, season) were associated with 25(OH)D levels in white Americans, but not in African Americans [14]. In the Adventist Health Study II, age, BMI, season, supplement use, total vitamin D intake, skin type, and sun exposure factors were significant predictors in white Americans, whereas only season, supplement use, and total vitamin D intake were predictors in African Americans [59]. In the Health ABC Study of elderly African Americans, significant predictors of 25(OH)D were similar to those in the present study: supplement use, dietary intake, BMI, walking, and season of blood draw, with supplement use being the strongest predictor [60].

Several other surveys have reported higher proportions of vitamin D deficiency among African American women than were observed in our study sample [61, 62]. The BWHS population is not representative of all African American women: in particular BMI is lower in the BWHS and use of female hormones is more common, and both would result in relatively higher levels of 25(OH)D. Nevertheless, the wide range of values in our sample permitted development of a prediction model with validation parameters on a par with those from prediction models in other populations. The internal validity of the work presented here would not be compromised by use of a sample that does not represent all women.

A limitation of the present study is the lack of granular data on vitamin D supplements. We did not collect data on the dose of the supplement. The prediction model might have been stronger if separate variables for low dose (e.g., 400 IU as part of a multivitamin) and high dose (e.g., 1000–2000 IU individual supplement) were included in the model. Nevertheless, supplement use accounted for 10 % of the variability in predicted vitamin D status in our study. Another potential limitation is lack of data on degree of skin pigmentation. However, two recent studies of skin pigmentation and 25(OH)D levels among several racial/ethnic groups found that measured constitutive skin color did not improve prediction of 25(OH)D concentrations when included in a model that had terms for race/ethnicity [63, 64].

Genetic variation may explain some of the interpersonal variation in 25(OH)D concentrations. We did not include genetic variants in the prediction model because there is a lack of consensus on which variants are associated with 25(OH)D in African Americans [8, 60, 65], and a previous study found that adding genetic variants to a 25(OH)D prediction model made little difference in explaining overall variation in serum 25(OH)D, especially in African Americans [65].

## Conclusions

In conclusion, data from this large prospective study suggest that African American women who have low predicted 25(OH)D have a greater risk of breast cancer relative to those who have sufficiently high levels. Vitamin D deficiency is common among African Americans. Indeed, of the 2856 BWHS participants who provided a blood sample in 2013–2015, 47 % had levels below 30 ng/mL (insufficient or deficient) and 22 % had levels below 20 ng/mL (deficient). If the present findings are confirmed in other prospective studies, preventing vitamin D deficiency may be an effective means of reducing breast cancer incidence in African American women.

## Abbreviations

1,25(OH)2D, 1,25-dihydroxyvitamin D; 25(OH)D, 25-hydroxyvitamin D; BMI, body mass index; BWHS, Black Women’s Health Study; CI, confidence interval; ER, estrogen receptor; IRR, incidence rate ratio; LC-MS/MS, liquid chromatography-tandem mass spectrometry; SD, standard deviation

## References

[1] Feldman D, Krishnan AV, Swami S, Giovannucci E, Feldman BJ (2014). The role of vitamin D in reducing cancer risk and progression. Nat Rev Cancer.

[2] Giovannucci E, Liu Y, Rimm EB, Hollis BW, Fuchs CS, Stampfer MJ, Willett WC (2006). Prospective study of predictors of vitamin D status and cancer incidence and mortality in men. J Natl Cancer Inst.

[3] Gorham ED, Garland CF, Garland FC, Grant WB, Mohr SB, Lipkin M, Newmark HL, Giovannucci E, Wei M, Holick MF (2007). Optimal vitamin D status for colorectal cancer prevention: a quantitative meta analysis. Am J Prev Med.

[4] Jacobs ET, Kohler LN, Kunihiro AG, Jurutka PW (2016). Vitamin D and colorectal, breast, and prostate cancers: a review of the epidemiological evidence. J Cancer.

[5] Eliassen AH, Spiegelman D, Hollis BW, Horst RL, Willett WC, Hankinson SE (2011). Plasma 25-hydroxyvitamin D and risk of breast cancer in the Nurses' Health Study II. Breast Cancer Res.

[6] Scarmo S, Afanasyeva Y, Lenner P, Koenig KL, Horst RL, Clendenen TV, Arslan AA, Chen Y, Hallmans G, Lundin E (2013). Circulating levels of 25-hydroxyvitamin D and risk of breast cancer: a nested case-control study. Breast Cancer Res.

[7] Kuhn T, Kaaks R, Becker S, Eomois PP, Clavel-Chapelon F, Kvaskoff M, Dossus L, Tjonneland A, Olsen A, Overvad K (2013). Plasma 25-hydroxyvitamin D and the risk of breast cancer in the European prospective investigation into cancer and nutrition: a nested case-control study. Int J Cancer.

[8] Yao S, Zirpoli G, Bovbjerg DH, Jandorf L, Hong CC, Zhao H, Sucheston LE, Tang L, Roberts M, Ciupak G (2012). Variants in the vitamin D pathway, serum levels of vitamin D, and estrogen receptor negative breast cancer among African-American women: a case-control study. Breast Cancer Res.

[9] Genkinger JM, Makambi KH, Palmer JR, Rosenberg L, Adams-Campbell LL (2013). Consumption of dairy and meat in relation to breast cancer risk in the Black Women's Health Study. Cancer Causes Control.

[10] Kim Y, Franke AA, Shvetsov YB, Wilkens LR, Cooney RV, Lurie G, Maskarinec G, Hernandez BY, Le Marchand L, Henderson BE (2014). Plasma 25-hydroxyvitamin D3 is associated with decreased risk of postmenopausal breast cancer in whites: a nested case-control study in the multiethnic cohort study. BMC Cancer.

[11] John EM, Schwartz GG, Koo J, Wang W, Ingles SA (2007). Sun exposure, vitamin D receptor gene polymorphisms, and breast cancer risk in a multiethnic population. Am J Epidemiol.

[12] Looker AC, Pfeiffer CM, Lacher DA, Schleicher RL, Picciano MF, Yetley EA (2008). Serum 25-hydroxyvitamin D status of the US population: 1988-1994 compared with 2000-2004. Am J Clin Nutr.

[13] Holick MF (1995). Environmental factors that influence the cutaneous production of vitamin D. Am J Clin Nutr.

[14] Freedman DM, Cahoon EK, Rajaraman P, Major JM, Doody MM, Alexander BH, Hoffbeck RW, Kimlin MG, Graubard BI, Linet MS (2013). Sunlight and other determinants of circulating 25-hydroxyvitamin D levels in black and white participants in a nationwide U.S. study. Am J Epidemiol.

[15] Kroll MH, Bi C, Garber CC, Kaufman HW, Liu D, Caston-Balderrama A, Zhang K, Clarke N, Xie M, Reitz RE (2015). Temporal relationship between vitamin D status and parathyroid hormone in the United States. PLoS One.

[16] Holick MF (2007). Vitamin D deficiency. N Engl J Med.

[17] Kumanyika SK, Mauger D, Mitchell DC, Phillips B, Smiciklas-Wright H, Palmer JR (2003). Relative validity of food frequency questionnaire nutrient estimates in the Black Women's Health Study. Ann Epidemiol.

[18] Furberg H, Millikan R, Dressler L, Newman B, Geradts J (2001). Tumor characteristics in African American and white women. Breast Cancer Res Treat.

[19] Gapstur SM, Dupuis J, Gann P, Collila S, Winchester DP (1996). Hormone receptor status of breast tumors in black, Hispanic, and non-Hispanic white women. An analysis of 13,239 cases. Cancer.

[20] Joslyn SA (2002). Racial differences in treatment and survival from early-stage breast carcinoma. Cancer.

[21] Palmer JR, Boggs DA, Wise LA, Ambrosone CB, Adams-Campbell LL, Rosenberg L (2011). Parity and lactation in relation to estrogen receptor negative breast cancer in African American women. Cancer Epidemiol Biomarkers Prev.

[22] Department of Health and Human Services. Center for Medicare and Medicaid Services. Regulation and Guidance. [https://www.cms.gov/Regulations-and-Guidance/Legislation/CLIA/Laboratory_Registry.html]. Accessed July 2016.

[23] College of American Pathologists. [http://www.cap.org/web/home/lab/accreditation]. Accessed July 2016.

[24] Chen H, McCoy LF, Schleicher RL, Pfeiffer CM (2008). Measurement of 25-hydroxyvitamin D3 (25OHD3) and 25-hydroxyvitamin D2 (25OHD2) in human serum using liquid chromatography-tandem mass spectrometry and its comparison to a radioimmunoassay method. Clin Chim Acta.

[25] Hojskov CS, Heickendorff L, Moller HJ (2010). High-throughput liquid-liquid extraction and LCMSMS assay for determination of circulating 25(OH) vitamin D3 and D2 in the routine clinical laboratory. Clin Chim Acta.

[26] Scotto J, Fears TR, Fraumeni JF, Schottenfeld D, Fraumeni JF (1996). Solar radiation. Cancer Epidemiology and Prevention.

[27] Hasite T, Tibshirani R, Friedman J (2009). The Elements of Statistical Learning.

[28] Kuhn M. Building Predictive Models in R Using the caret Package. J Stat Softw. 2008;28(5):26.

[29] Willett WC (1998). Nutritional Epidemiology.

[30] Hu FB, Stampfer MJ, Rimm E, Ascherio A, Rosner BA, Spiegelman D, Willett WC (1999). Dietary fat and coronary heart disease: a comparison of approaches for adjusting for total energy intake and modeling repeated dietary measurements. Am J Epidemiol.

[31] Joh HK, Giovannucci EL, Bertrand KA, Lim S, Cho E (2013). Predicted plasma 25-hydroxyvitamin D and risk of renal cell cancer. J Natl Cancer Inst.

[32] Jung S, Qian ZR, Yamauchi M, Bertrand KA, Fitzgerald KC, Inamura K, Kim SA, Mima K, Sukawa Y, Zhang X (2014). Predicted 25(OH)D score and colorectal cancer risk according to vitamin D receptor expression. Cancer Epidemiol Biomarkers Prev.

[33] Bertrand KA, Giovannucci E, Liu Y, Malspeis S, Eliassen AH, Wu K, Holmes MD, Laden F, Feskanich D (2012). Determinants of plasma 25-hydroxyvitamin D and development of prediction models in three US cohorts. Br J Nutr.

[34] Holick MF, Binkley NC, Bischoff-Ferrari HA, Gordon CM, Hanley DA, Heaney RP, Murad MH, Weaver CM, Endocrine S (2011). Evaluation, treatment, and prevention of vitamin D deficiency: an Endocrine Society clinical practice guideline. J Clin Endocrinol Metab.

[35] Gandini S, Boniol M, Haukka J, Byrnes G, Cox B, Sneyd MJ, Mullie P, Autier P (2011). Meta-analysis of observational studies of serum 25-hydroxyvitamin D levels and colorectal, breast and prostate cancer and colorectal adenoma. Int J Cancer.

[36] Yin L, Grandi N, Raum E, Haug U, Arndt V, Brenner H (2010). Meta-analysis: serum vitamin D and breast cancer risk. Eur J Cancer.

[37] Engel P, Fagherazzi G, Boutten A, Dupre T, Mesrine S, Boutron-Ruault MC, Clavel-Chapelon F (2010). Serum 25(OH) vitamin D and risk of breast cancer: a nested case-control study from the French E3N cohort. Cancer Epidemiol Biomarkers Prev.

[38] Bertone-Johnson ER, Chen WY, Holick MF, Hollis BW, Colditz GA, Willett WC, Hankinson SE (2005). Plasma 25-hydroxyvitamin D and 1,25-dihydroxyvitamin D and risk of breast cancer. Cancer Epidemiol Biomarkers Prev.

[39] Freedman DM, Chang SC, Falk RT, Purdue MP, Huang WY, McCarty CA, Hollis BW, Graubard BI, Berg CD, Ziegler RG (2008). Serum levels of vitamin D metabolites and breast cancer risk in the prostate, lung, colorectal, and ovarian cancer screening trial. Cancer Epidemiol Biomarkers Prev.

[40] McCullough ML, Stevens VL, Patel R, Jacobs EJ, Bain EB, Horst RL, Gapstur SM, Thun MJ, Calle EE (2009). Serum 25-hydroxyvitamin D concentrations and postmenopausal breast cancer risk: a nested case control study in the Cancer Prevention Study-II Nutrition Cohort. Breast Cancer Res.

[41] Krishnan AV, Swami S, Feldman D (2010). Vitamin D and breast cancer: inhibition of estrogen synthesis and signaling. J Steroid Biochem Mol Biol.

[42] Stoica A, Saceda M, Fakhro A, Solomon HB, Fenster BD, Martin MB (1999). Regulation of estrogen receptor-alpha gene expression by 1, 25-dihydroxyvitamin D in MCF-7 cells. J Cell Biochem.

[43] Ameri P, Giusti A, Boschetti M, Murialdo G, Minuto F, Ferone D (2013). Interactions between vitamin D and IGF-I: from physiology to clinical practice. Clin Endocrinol (Oxf).

[44] Yao S, Sucheston LE, Millen AE, Johnson CS, Trump DL, Nesline MK, Davis W, Hong CC, McCann SE, Hwang H (2011). Pretreatment serum concentrations of 25-hydroxyvitamin D and breast cancer prognostic characteristics: a case-control and a case-series study. PLoS One.

[45] Abbas S, Chang-Claude J, Linseisen J (2009). Plasma 25-hydroxyvitamin D and premenopausal breast cancer risk in a German case-control study. Int J Cancer.

[46] Wootton AM (2005). Improving the measurement of 25-hydroxyvitamin D. Clin Biochem Rev.

[47] Sonderman JS, Munro HM, Blot WJ, Signorello LB (2012). Reproducibility of serum 25-hydroxyvitamin d and vitamin D-binding protein levels over time in a prospective cohort study of black and white adults. Am J Epidemiol.

[48] Shin MH, Holmes MD, Hankinson SE, Wu K, Colditz GA, Willett WC (2002). Intake of dairy products, calcium, and vitamin d and risk of breast cancer. J Natl Cancer Inst.

[49] Robien K, Cutler GJ, Lazovich D (2007). Vitamin D intake and breast cancer risk in postmenopausal women: the Iowa Women's Health Study. Cancer Causes Control.

[50] Cadeau C, Fournier A, Mesrine S, Clavel-Chapelon F, Fagherazzi G, Boutron-Ruault MC (2015). Interaction between current vitamin D supplementation and menopausal hormone therapy use on breast cancer risk: evidence from the E3N cohort. Am J Clin Nutr.

[51] McCullough ML, Rodriguez C, Diver WR, Feigelson HS, Stevens VL, Thun MJ, Calle EE (2005). Dairy, calcium, and vitamin D intake and postmenopausal breast cancer risk in the Cancer Prevention Study II Nutrition Cohort. Cancer Epidemiol Biomarkers Prev.

[52] Chlebowski RT, Johnson KC, Kooperberg C, Pettinger M, Wactawski-Wende J, Rohan T, Rossouw J, Lane D, O'Sullivan MJ, Yasmeen S (2008). Calcium plus vitamin D supplementation and the risk of breast cancer. J Natl Cancer Inst.

[53] Kuper H, Yang L, Sandin S, Lof M, Adami HO, Weiderpass E (2009). Prospective study of solar exposure, dietary vitamin D intake, and risk of breast cancer among middle-aged women. Cancer Epidemiol Biomarkers Prev.

[54] Engel P, Fagherazzi G, Mesrine S, Boutron-Ruault MC, Clavel-Chapelon F (2011). Joint effects of dietary vitamin D and sun exposure on breast cancer risk: results from the French E3N cohort. Cancer Epidemiol Biomarkers Prev.

[55] Edvardsen K, Veierod MB, Brustad M, Braaten T, Engelsen O, Lund E (2011). Vitamin D-effective solar UV radiation, dietary vitamin D and breast cancer risk. Int J Cancer.

[56] Abbas S, Linseisen J, Rohrmann S, Chang-Claude J, Peeters PH, Engel P, Brustad M, Lund E, Skeie G, Olsen A (2013). Dietary intake of vitamin D and calcium and breast cancer risk in the European Prospective Investigation into Cancer and Nutrition. Nutr Cancer.

[57] Redaniel MT, Gardner MP, Martin RM, Jeffreys M (2014). The association of vitamin D supplementation with the risk of cancer in postmenopausal women. Cancer Causes Control.

[58] Bjorn Jensen C, Thorne-Lyman AL, Vadgard Hansen L, Strom M, Odgaard Nielsen N, Cohen A, Olsen SF (2013). Development and validation of a vitamin D status prediction model in Danish pregnant women: a study of the Danish National Birth Cohort. PLoS One.

[59] Chan J, Jaceldo-Siegl K, Fraser GE (2010). Determinants of serum 25 hydroxyvitamin D levels in a nationwide cohort of blacks and non-Hispanic whites. Cancer Causes Control.

[60] Hansen JG, Tang W, Hootman KC, Brannon PM, Houston DK, Kritchevsky SB, Harris TB, Garcia M, Lohman K, Liu Y (2015). Genetic and environmental factors are associated with serum 25-hydroxyvitamin D concentrations in older African Americans. J Nutr.

[61] Looker AC, Dawson-Hughes B, Calvo MS, Gunter EW, Sahyoun NR (2002). Serum 25-hydroxyvitamin D status of adolescents and adults in two seasonal subpopulations from NHANES III. Bone.

[62] Harris SS, Dawson-Hughes B (1998). Seasonal changes in plasma 25-hydroxyvitamin D concentrations of young American black and white women. Am J Clin Nutr.

[63] Au LE, Harris SS, Dwyer JT, Jacques PF, Sacheck JM (2014). Association of serum 25-hydroxyvitamin D with race/ethnicity and constitutive skin color in urban schoolchildren. J Pediatr Endocrinol Metab.

[64] Nessvi S, Johansson L, Jopson J, Stewart A, Reeder A, McKenzie R, Scragg RK (2011). Association of 25-hydroxyvitamin D3)levels in adult New Zealanders with ethnicity, skin color and self-reported skin sensitivity to sun exposure. Photochem Photobiol.

[65] Batai K, Murphy AB, Shah E, Ruden M, Newsome J, Agate S, Dixon MA, Chen HY, Deane LA, Hollowell CM (2014). Common vitamin D pathway gene variants reveal contrasting effects on serum vitamin D levels in African Americans and European Americans. Hum Genet.

